# Babies in therapy, psychoanalytic interventions for infants and their parents

**DOI:** 10.3389/fpsyt.2022.1054372

**Published:** 2022-11-23

**Authors:** Christine Anzieu-Premmereur

**Affiliations:** Psychiatry Department, Columbia University, New York City, NY, United States

**Keywords:** psychoanalysis, depression, primitive anxieties, dyadic therapy, playfulness

## Abstract

**Babies in therapy:** The study “Psychoanalytic interventions for infants and their parents” conducted on infants through the lens of psychoanalysis and clinical work with both parents and infants all contribute to our knowledge of the nature of early relationship disorders. Psychoanalytic theory's concepts of the depressive position, early defense mechanisms, transference, and psychosomatic reactions to depressive emotions are shown to be crucial in clinical cases, giving therapists new tools for intervention and increasing efficiency. Psychoanalysts have researched the long-lasting effects of early disappointments and the sense of being helplessly abandoned; they emphasize that a disruption in the relationship with the caregiver can produce a psychic economy oriented on the avoidance of anxiety, leaving less energy for development. New parents and their sick infants can benefit from early therapies with therapeutic potential and the possibility of preventing future narcissistic pain issues if they are based on psychoanalytic thinking and knowledge of early symptoms.

## Introduction

Clinical work with parents and babies, psychoanalytic observation of infants, and studies of infancy all contribute to our understanding of how early relationship disorders develop. This paper focuses on the infant's inner world and how some classical and modern Freudian concepts can help the analyst working with dyads. These ideas range from the presumption of primary narcissism to the drive theory, which links libido development to the need for self-preservation and the resulting tensions, conflicts, anxieties, and defense mechanisms. The baby's primitive internal development is understood to be a complex unconscious dynamic process.

The profound effect of early disappointments and the feeling of being helplessly abandoned has been studied by many psychoanalysts, who emphasize that a disruption in the relationship with the caregiver can cause a psychic economy oriented on the avoidance of anxiety, leaving less energy for development.

Joint therapies provide both the suffering mother and child with support in dealing with each other's narcissism; depressive meltdowns in toddlers are always attacks on the integrity of the self that can have serious developmental consequences. For this reason, early and rapid intervention is essential when signs of concern are observed in toddlers, but it is still difficult to know the long-term influence of such interventions. Primary breakdowns have far-reaching effects on people's lives, from early childhood disorders, such as developmental delays or attention difficulties, to serious obstacles to accessing the oedipal conflict, damage in adolescence to traumatic recurrences in adulthood. Even if they fully recover after receiving therapy, some people will still have a problem with the motor and behavioral discharge, cutting off the transitional stakes necessary to sustain the preconscious functioning that would allow for a cognitive activity to form. From the first few months of life, there are disturbing behavioral patterns. I will talk about situations in which libidinal movements are not invested and the risks they pose to the development of the subject.

The narcissistic wound the child feels poses a threat of primary depression, whether due to the mother's postpartum depression, difficulties with the child's attachment, or the baby's difficult temperament.

## Lack of vitality and depressive reactions in infancy

At the beginning of life, these wounds leave indelible marks: distress, collapse, the feeling of being let down, of falling vertiginously without hope, the feeling of spreading, of liquefying without contact. These feelings of despair, inscribed in the body as much as in the psyche, are sources of narcissistic damage that can sometimes be repaired.

According to the axis of depressed posture, infant depression can be classified into two clinical entities.

The depressive position described by Melanie Klein (1) is a normal process in the infant's development: it is a stage from which the child enters a relationship with an object and considers himself independent and distinct from his mother. If the threat is the loss of this separate object, which is accompanied by feelings of ambivalence and guilt, it is also the first step to creating a stable and permanent internal object, even in its absence.

During the establishment of this depressive position, the pain of the loss may be too acute and trigger the manic defense. Afterward, the problem of loss and separation, with sadness and mourning, is in the foreground. This is a time when we can share with the child an intermediate space where we are two distinct and empathetic individuals. The therapist will act as an impartial third party by allowing the mobility of the fantasmatic representations through plays, interpretations, and comedy, which encourage libido circulation.

Because of the narcissistic nature of this investment, such as the nipple in the mouth is a part of the self, the loss is first experienced as a loss of a partial object, such as the breast or the oral sensory experience of being with the mother, which is felt as catastrophic and irretrievable. Before experiencing depressive effects, infants experience terror, the anguish of annihilation, the suffering of being able to only survive without being lively, and psychosomatic disorganization. The stake is then the constitution of an identity, a continuous, stable, and well-organized Ego, which can accommodate various perceptions.

As seen in his “Still Face” experiment from 1975, in which he had mothers of typically developing 3-month-olds maintain a rigid and expressionless expression, Tronick was able to observe the baby's expression of helplessness, a depression in the making (2):

The baby looks at his mother and smiles. The mother remains impassive. The baby intensifies his look, stretches his arms, and frowns. Then, the baby turns away for a few seconds and returns toward the still-frozen mother. Faced with the failure of its attempts at interaction, the baby tires, yawns, looks at its mother, and “ends up curling up in an attitude of powerlessness, face turned away, body motionless.” This interaction does not last more than 3 min and shows the infant's vulnerability. Mothers interviewed after this experience reported how much they experienced their child's agitation and anxiety with a feeling of sadness, anger, and despair.

I quote Winnicott (3): “A slight defect in holding...gives the child a sense of endless falling.”

“The depressive position, classically, opens on the mourning of the omnipotence to have its needs satisfied, on the renunciation of the desire without limits... Partial renunciation, we all know it well! But before being dispossessed of the fantasy of omnipotence, it is still necessary to have experienced it!” As demonstrated by Winnicott, object relations are established in the first few months of life. These ties are founded on the child's awareness of a world outside themselves in which they can take action and engage in reciprocal trade. The mother maintains the illusion that the child has unlimited power over the world. The child's belief in their own agency and cognitive capacity is undermined by this narcissistic involvement in the world as a source of gratification. This belief is essential to developing thought in real life. Therefore, at the start of life, building a strong connection to the real, physical world is essential.

Maternal care's inevitable failures and discontinuities are always felt as narcissistic losses. The child learns that the maternal container has its faults and limits, which are real threats to an infant easily deprived of its state of original distress. Observations of babies with their mothers show how much most of the defects of mutual adjustment can be corrected and how much the dyads know how to reconcile after a misunderstanding. The adjustment between needs and satisfactions, desires and gratifications is never perfect. The mother fails because of too much anticipation or too much delay. She can only be “good enough.” Disappointments and unmet expectations, as well as tensions and conflicts stemming from the child's inherent tendency toward individuation, are woven into the fabric of the child's daily life. The maternal excitability barrier is always partial. These recurrences can have a cumulative effect and take on significant traumatic value.

Depressive anxieties are frequent and transient in babies, as observations of infants at home clearly show. They are generally repaired by the quality of the psychic and physical holding. However, unpleasant reactions to smiling, making eye contact, psychosomatic disorders, and withdrawal symptoms might develop if this bonding experience is lacking, whether from the mother or from the child.

In the very first months of life, the child does not suffer from losing an object. The relationship is still too fragile. An infant is in the slow process of differentiating between self and object, and the quality of the object's investment, which is typically still precarious, will be affected.

To establish a solid connection, it is crucial to have frequent sessions. The so-called anaclitic depression prevents children from engaging in sufficient cerebral stimulation. These solemn infants, who never smile and lack any vital tonus, are typically stuck in the infernal repetition of self-calming actions, signaling a transition from autoerotic games to functioning. As Bowlby (4) and Winnicott (3) pointed out, each in their own way, it is not the separation that leads to the baby's depression; it is the loss of hope. An object that has disappeared and been rendered inaccessible during rapid weaning, for example, an interaction with an inanimate mother without vocalizations or looks, or an illness or a surgical operation, can suppress the relationships with the object and leave the body and the psyche destitute. The infant is atonic, withdrawn, slowed down, and rapidly disorganized on the psychosomatic level and can present the pathological defenses described by Selma Fraiberg (5): the avoidance of eye contact, the paralysis of behavior or freezing, and the transformation of effects when, instead of manifesting distress or anxiety, an abused baby manifests a noisy joy and a disorganized excitement, which is called self-aggression.

Between birth and 6 months of age, a baby's “depressive” behaviors are more a response to the collapse of the tonus of life, to the loss of the possibility of investing, than to the loss of an object that is not yet solidly established. As we hold him in our arms, we see a newborn who has lost his skills, isolated from others and himself, with a frozen face, without laughing or smiling, without joyful expression in the eyes; he is rather indifferent, rarely surprised, who turns away and vocalizes very little (6). Illness, pain, and fatigue create these reactions and the loss of relationships between internal objects. At the beginning of life, the body envelope is still fragile, and to maintain the sensations and the experience of being alive through movements, the body's action is essential. The contacts with others help to constitute the physical unity of the body and the being. The feeling of being held, the touch, and the glances all leave physical and emotional imprints. A failure of the object's constitution is a failure of the subjects' constitution. Reestablishing a corporal dialogue is an emergency (7).

It is the economic aspect of the maternal psychic functioning that is communicated to the child in the dance of the preverbal games. The prosody, the rhythm of the enunciation as well as of the carrying, the intensity of the glances as well as the voice are essential signals to which the baby answers with his own register of primitive impulsiveness (8).

We cannot ignore, however, that depression does not exist without hatred; it is an early rage rather than an organized emotion, as Winnicott (3) shows. Let us not forget that Freud classified indifference as a special case of hatred. This intense and early development of negative movements requires an active mobilization of life movements on the part of the entourage. Early therapies are often emergencies.

The impact of intersubjectivity on the psychic development of the child and on the genesis of the sense of self, the first foundation of the Ego, is essential and can provide reference points to the analyst who works with infants. Intervening directly with a baby when the mother allows access to her child is immediately effective. If we think that the emergence of the first representations and the establishment of the processes of symbolization take place in interactive play, we can help a mother give the baby bodily anchoring of the experiences they share and pay attention to the sensations that the baby experiences without always being able to integrate them. The extraordinary appetite of babies for contact with others always facilitates the therapist's work. At the beginning of life, play is gestural. As such, it lays the groundwork for the child's future communication options and is crucial for developing emotional and affective sharing.

Primary depression is always associated with early trauma. When one's own survival is at risk, Childhood sexuality and its auto and hetero erotic investments are no longer appropriate. The risk is the libidinal loss, the loss of the quality of the libido connection, hence a desexualization of the psychic functioning. The loss of pleasure from autoerotic investment and the loss of the joy of a successful action are all possibilities if one's libido declines or if the quality of one's libido connection deteriorates.

## The role of the capability for representation

If a nursing infant does not recognize himself in her mother's eyes, they will not be able to share in any joy together and will break the bond that has kept them connected to their mother's pleasure all along. There is a heterogeneity in the depression of infants and older children. In the beginning, the atony of investments dominates the reactions of withdrawal as an ultimate defense of self-preservation. It is in the depression of death that Palacio Espasa (9) observed how the relationships and the binders would disappear when the loss of a representation of the object is experienced as a catastrophe: losing the sense of oneself. A failure in the meeting with the mother is accompanied by a lack of memory trace of her, an absence of representation; hence, the ability to invest in an object is gravely compromised.

Winnicott (3) was the first to draw attention to the depressive suffering of what he called “psychotic depression,” which occurs when the loss of an object is accompanied by the loss of a part of the self in a bodily experience: loss of the breast, loss of the mouth, a loss that cannot be elaborated into a psychic experience, leaving the subject destitute and in a state of helplessness.

In psychoanalytic therapies, the goal is to restore the dyadic creative drive through which the infant receives from the mother's skin the senses of smell, taste, warmth, color, security, and maintenance between loving and erotic attachments. A mother with the genius to collect her child's experiences into a cohesive whole will provide the impression of one's own emotional experience. Let's look at the first smiles of babies when they address others: how much the baby seeks to make contact and to seduce.

The feeling of continuity and permanence of the self, what Balint (10) called “fundamental confidence,” is a framework that supports narcissism, giving it its qualities of confidence and quietude or distrust and greed. This feeling of internal security depends on the primary relationship to the object, the quality of the pleasure taken in the exchange, and what is appropriate in autoeroticism. Sucking in anticipation of breastfeeding is proof of this confidence in oneself and the environment. Active motions at taking in are essential to feeding and tolerating helplessness and passivity. As soon as the environment lacks that support, powerlessness is felt, and the pleasure of desire disappears. A defensive system is then set up, where the threatened ego immediately solicits aggressiveness in an attempt to safeguard the limits of the ego actively. The therapeutic response is to make a narcissistic alliance that counterbalances the insecurity to make the relationship viable and tolerable: it is necessary to reanimate, to reactivate the movements of life and linkages in the transference, and to restore the ego and its capacity for confidence.

In the danger of de-objectalization, offering a psychic and physical envelope, firm and flexible, is essential: it would be an introjectable container for the baby, provided that the rhythmicity of the tuning at the time of the care allows a true gathering of the senses, a transforming action of the impulsive flow as Bullinger (11) proposes. This shows the importance of direct interventions with infants whose mothers interact inadequately.

The therapeutic process modifies the psychic economy. The therapist's game engages and mobilizes the infant's interest, keeps his body active, and stimulates the development of the related fantasy.

The triangulation associated with the presence of the therapist in front of the dyad, or the mobilization of the paternal presence at the insistent invitation of the analyst, modifies the libidinal investment of the baby for a “non-mother” character who has an essential anti-depressive function by modifying the constellation of anxieties and defenses that characterizes the 6-month-old child at the moment when he reaches the depressive position. The father builds a bridge between the mother and the kid; The father, not as another maternal object, but as different, and containing his wife, creates a bridge between her and the child; it is he who allows the establishment of links.

Using an outside authority figure, such as a therapist who acts as a father figure, is cathartic because it releases pent-up libidinal energy, makes room for fresh investments, and kick-starts stagnant development stifled by the narcissistic system (12).

## The role of play

The great play models are organized the absence: the fort da the hide-and-seek are games of controlled loss. The discovery and creation of games, pleasures of all kinds, and the sexualization of life always show an improvement in babies; the appearance of a smile in impassive infants and laughter in little ones who have finally become enthusiastic are reliable signs of change (13).

Stimulated by the environment, babies begin to laugh and become happy and excited at the age of 4 months. By the end of the first year, children begin to laugh on their own *via* their own actions and decisions, and it is from 18 months on that a real sense of humor (non-verbal) will emerge in incongruous and surprising situations.

I am not talking here about the laughter that relieves tension and has poorly organized effects or the big laughs of manic defense; I am referring to the association of pleasure and control in the sense of humor.

### A 3-month-old baby

Anna is an almost 3-month-old girl, sullen and silent. Her mother is convinced that she will become autistic because she makes no sounds and smiles little. I know this mother well. She has had disability anxiety for each of her previous children and had to undergo a therapeutic abortion before becoming pregnant with Anna. The mother-daughter encounters are quite gentle around breastfeeding, but the exchanges of glances are poor and lost in anguish. I address the baby directly, telling her how her mother wanted a wonderful little girl but had lost confidence since the tragedy of the abortion, and I add, in a deliberately theatrical tone, that her mother is a woman of inordinate demands who wants perfect children; I surprise the mother, who starts laughing when I add that she has all the possible faults of a mother who idealizes the maternal function. I know that Anna is smiling with her father, and I tell her what a conflict it must be for the little girl to have a handsome father and an angry mother. Anna's mother, of course, reacts strongly and addresses her daughter in turn, saying how much she loves and values her baby, and her voice changes, becoming softer and more melodious. Anna turns to me and coos, then make eye contact with her mother and gets lost in her eyes, smiling. She then not stops babbling and becomes a very happy baby in a few weeks.

It is more difficult when the baby must face the negative effects of a rejecting mother. The threats of annihilation of the internal world are then intense. The persecutory experience is constant. These babies present obvious mood disorders. They are apathetic, inhibited, and always serious. Their development is restricted; attachment disorders take the lead very quickly, with a deficiency in bonds; these children can be entrusted to any stranger. They are neither fierce nor wild. The mother has not been able to be a mirror image of the child's emotions and has not been able to invest in the child as an object of love (14). The narcissistic damage is inevitable, as is the accompanying sense of guilt for having caused the argument with one's mother and a general inability to find peace in the world.

For a child's mind to develop, there must be something around that can hold it, comfort it through its pains, and challenge it just enough. Like a broken container, an uninteresting cosmos might trigger primal fears of abandonment and isolation. The excess of depressive suffering does not allow for the continuity and stability of the psychic structure; the introjective capacities of the baby are diminished; the developmental potentials cannot be realized and remain frozen in pathological identifications (15). The primary identification is with an object that ensures vital functions in a harmonious relationship, and this relationship allows psychic growth. Primary depression can be the equivalent of psychic death with proper defenses in place.

The baby has psychically withdrawn from the relationship. He has disengaged himself.

This failure in constructing the subject as an object of desire jeopardizes the construction of the fantasy. These sick children do not play or laugh.

The feeling of “existing” remains extremely fragile in children with early narcissistic disorders. The feeling of security is sometimes regained by looking at them and through rhythmic exchanges. Eye-to-eye contact is a tactile experience for infants. When a mother can, during therapy with her baby, rediscover the pleasure of contact with a baby she is rocking and singing a nursery rhyme to, a common skin is put back in place (8).

### An inconsolable 3-year-old boy

A 3-year-old boy I had known as a baby, subjected to the disorganizing anxieties of his borderline mother, was subject to inconsolable terrors. During the session, he drew elephants covered with three layers of skin. I pointed out to him that they must have felt well wrapped up, unlike him. He described his anxiety about dissolving into the water like sugar on the way to the pool or spilling like an inconsistent liquid on the way to bed at night. This child had never laughed, and it took several years of therapy for him to find a sense of humor that makes distress funny.

In early therapy, play fulfills several functions, from putting the mother's relational capacities back into play to engaging in eye-to-eye communication with a baby who is just waiting for it to the slow resuscitation of a withdrawn infant, hypotonic and almost desireless. A game of imitations, tuning, sound and visual mirroring, offering empathy, sympathy, and the ability to read effects (16).

The emotional exchange is the relationship between the internal world and the outside, the material that allows the relationship to be created and to continue. The danger is not so much hatred as withdrawal, non-emotion, as Bion (17) calls “anti-emotion.”

It is essential to allow a baby to act in the world and feel capable of acting on his own body and others. The game unfolds with a shared pleasure, the amused complicity of the therapist. It is important to remain naïve and enthusiastic enough to be surprised by the baby and its formidable capacities for relationship and recovery.

As Lore Schacht (18) has shown, wonder and being surprised are part of therapy with children. It is a capacity that is quickly lost in depression. Emotional sharing is the benchmark for the therapist, sometimes mired in discouraging affect, sometimes excited by the baby's rapid engagement.

Play allows the object's response to be represented: what has been refused or has been impossible, the prohibitions and the losses; play also allows the child to satisfy its need for action and transformation. The therapist must try to remain creative to keep a sense of humor, which allows the differentiation between internal and external reality to be maintained while playing for real or fictitiously. We stay in the realm of illusion to give the baby ways to represent things and to give him a chance to understand how symbolization works.

How fix the damage to the object? It is all the work of the depressive position. Moreover, in the playground that the therapist offers to the child by addressing him directly, a whole chain of representations can develop that manifest the dynamics and the imbalance linked to the depressive symptoms.

I will present my first encounter with a depressed family whose first child compulsively pulls out his hair. This is an example of a way of intervening with analytical thinking based on Freudian metapsychology, the internal economic aspect of the psychic apparatus, and the freedom to play that Winnicott gives.

## A toddler suffering from a compulsion to pull out her hair

Paul walks embarrassed, badly balanced on his small, spindly legs. He is alone in his discomfort. His parents consider him an independent person who does not need support.

His father bursts out laughing loudly at seeing him so awkward and frightened when he enters my office. The whole family seems caught up in the fear of the analyst in a stranger's anxiety, leaving the tiny boy in hyper-control of his wobbly body, looking hard. His mother is more worried about my gaze than her son, who will reject her brutally when she finally extends her hand. Paul falls, gets up quickly, and rushes to the toys on my desk without exchanging a glance.

He is a little boy who does not smile and has a stern look and a bitter mouth. He has almost no hair, his skin is damaged, and his appearance is strange and disharmonious. Paul has been compulsively pulling out his hair for several months.

According to his parents, this is a reaction to frustration, but when I try to connect the dots with events, they are surprised to discover that this symptom began when the baby moved to a daycare center.

The father reports that he is furious at his son's reaction and tends to scream and sometimes grab his arm violently, as his own father did when he was holding himself badly. He can talk about this abuse with legitimacy, thus leaning on his father's model, even if this admission is a request for help on his part.

Paul's mother cannot tolerate dirt, and the falling hair drives her crazy and makes her abusive, forcing her to deny the child's suffering.

Paul walks over to my desk, stubbornly avoiding his parents and clinging to a piece of furniture. A few stuffed animals attract his attention. He is more interested in the concrete aspect of the object than in its symbolic value.

His body is solidified around a clumsy muscular carapace, a defensive second skin, as Esther Bick (19) has shown. He is not harmonious because of this muscular contracture. This little boy is not attractive.

One scenario that comes to mind is that of immature, emotionally stunted parents who ignore or abuse their infant. According to Ferenczi et al. (20), when a child is not welcomed he is subject to depression and destructiveness.

Paul touches the toys without looking at them while his parents repeat: “Say thank you to the doctor, do not touch, apologize.”

Conversely, the inability to assign this little child a generational position is a manifestation of the restriction against touching, which is one of the organizers of oedipal prohibitions. A child is not touched by his parents if he is not in pain. He is repressed in his attempt at action and out of curiosity. No creativity.

When Paul is left alone with a stranger, he tries to take his anger out on his toys, but his parents, who have a very strong superego, stop him.

I think the symptom of pulling out hair is associated with a lack of sensation between mother and child, with the absence of a container and solid holding that can be internalized. This brings Didier Anzieu's Moi Peau to mind (21).

Their fear of judgment is extreme. A terrifying superego dominates the atmosphere, inhibiting everyone (22). As I point out, Paul's mother recognizes how impossible it is for her to play with her son. When they are together, she cannot stand the needs of the child who touches everything.

I say how distressed the presence of her baby makes her feel. This intervention leads to a respite from parental prohibitions, and Paul grabs a wooden duck. He places it on my desk, and the toy falls on the floor. She holds her breath, visibly distressed.

I then take the duck back and put it on the table, which I tap with the flat of my hand, saying, “Bad duck!” Paul opens his eyes, looks at me at last and waits for my reaction. I repeat the gesture of tapping the table while talking to the duck. Paul opens his mouth, drops his arms on his legs, and looks at me with a burst of surprise. The muscular relaxation is impressive. His mechanical robot look is gone. He approaches the table, throws the duck on the floor and solicits my intervention with his eyes. I repeat, “naughty duck!” We start a repetitive game, where he throws the duck more and more rigorously.

The persecutory atmosphere of the session has dissipated. Paul slams the table as he throws the duck on the floor and screams with his mouth open and head tilted back; it is more of a scream than an expression of joy, but his mother immediately interprets it as a laugh. She exclaims, “My son has never laughed!” This very brief outburst is followed by an excited lively look and a silent request to play again, which we do, with all the toys in his hand.

Paul's father is shocked that I would allow this transgression, but since the change in their son moves his wife, he agrees that I should say, “It's a game!”

Paul, however, does not smile and does not babble. But his look has become alive, and he solicits me. At the end of our first meeting, he points out a small plush ball, and I let him take this toy, which he will take home, while I ask the parents to think about bringing back the toy at the time of our second appointment, which they did, explaining their surprise as Paul replayed the fallen animal and the tapped table at every opportunity. They accepted that it was a game and were impressed by their son's ability to remember the session. This was their first positive comment about Paul. But the child pulls his hair out every night at home, and the parents' screaming does not help.

As I picked up this family in the waiting room for the second appointment, I was amazed to see this lone toddler sitting lopsidedly on the couch, holding a bottle in his mouth, with a blank stare, while both parents were sitting farther back in armchairs, busy with their own reading.

After a reunion with Paul, who took a while to exchange a glance, he got excited and repeated the game of the bad duck. I say my question is about the child's solitude with his bottle. The parents are proud to have an independent baby because, since the age of 6 months, Paul has held his bottle alone; they thought he no longer needed any contact with them.

I think of the relationship between the child's muscular contraction, aggressive tension, and maternal abandonment situations. Paul's mother understands my problem and asks for an interview with me alone. Working with the parents will allow for changes in their closeness to their baby's body and primary needs.

The discharges of tension and aggression will playfully continue in Paul. We play with balls that we exchange while I evoke the movements of proximity and distance from the maternal object.

After several weeks of guidance from the parents and playing with Paul, the symptom of hair-pulling changed. As soon as he pulls out his hair, her mother now sits next to him and gradually offers him tender contact. A doll was found in my office, whose large soft hair has become a subject of pleasure to caress and is on the way to becoming a fetish object.

But Paul needs to find self-calming actions and move from discharge to bonding. The child's therapy will last for a few years. Paul was an infant suffering from emotional neglect and a lack of auto-erotic capacity, and when he became a toddler addicted to a painful, compulsive discharge of despair and aggression. He then developed an ability to play when his mother found pleasure in interacting with him in a containing and daydreaming way (23).

Paul was a child “beyond the pleasure principle” (24), fed with bottles without the presence of the maternal object, and raised in silence when speech only served to emit prohibitions. The impulsive needs were ignored or restrained. Only the discharge remained as a solution to the tension. The experience of pleasure requires the presence of the object and the possibility of satisfaction Connivance around pleasure is sharing in the mirror that a mother may provide by acting as an accomplice and acknowledging the child's desires.

The complicity developed with the analyst allowed for exchanges of glances, emotions, then laughter. It is one of the essential roles of the third party to provide the necessary elements for satisfaction (13).

By identifying his son as being capable of joy, Paul's mother began to find pleasure in playing with him. As a mother abandoned by her own mother, she expected her son to be the one who supported and loved her.

Paul's feelings have been given substance by my interpretations of them. The ability to welcome satisfaction, enjoyment, surprise, and discoveries are balanced—or not—with the capacity for adaptation and control, which has a value of economic balance. The analyst's efforts help the kid develop the skills she needs to overcome the protective mechanisms that hold her back, such as the non-mentalized discharge processes and short circuits that deplete the richness of the child's imaginative play and her ability to represent the world around her.

Paul's first laugh in the session was a mixture of brutality and fragility. Subjected to the calming and destructive process of hair pulling, in an undifferentiated sensuality, he remained attached to the perceptive elements of the relationship. It is indeed an index of the self's premature reaction to the early trauma. By channeling their excitement into positive physiological ego sensations and the excitatory function of the analyst, who speaks and encourages the regression as access to passivity, the young child was able to feel a positive effect. Anguish is sometimes soluble in laughter.

The effect of surprise plays an essential role here. As Winnicott (25) shows in the use of the squiggle, it is when the patient surprises himself that a persistent therapeutic action can take place: the discovery of the unknown of the unconscious never before experienced. “The ability to surprise oneself presupposes the security of being carried by the framework, by the transferential relationship; it presupposes experiencing oneself as being in the attention and solicitude of the person in front of the other” [(26), p. 211].

### The social laughter

I observed Paul at the nursery when he was two and a half years old: he ran straight ahead without looking at his mother, who was leaving, made a tour of the friends who had already arrived, settled in the middle, and burst out laughing while shaking his head. A wild, forced laugh was the signal for the group: all laugh and approach him. They shook their heads in rhythm and laughed out loud. Paul forced himself to laugh again to maintain the general atmosphere and the feeling of being together. His mother was gone, and the group was here. With each difficult transition, he shook his head and forced a not-so-funny but convincing throat laugh to trigger a manic defense signal. Merging with the group of restless laughers allowed him to get through the transition without being alone.

The predictable emergence of a discharge of both anxious tension and excitement, the laughter of the small child is contagious. The narcissistic contribution is immediate: “I exist. The others imitate me and surround me.” The vitality of the ego-body associated with the laughing gesticulation gives the pleasure of being in the center of the group explosion.

For Paul's laughter to take on an auto-erotic value, it took many sessions of repeating the actions of throwing and tapping and then putting the toys in a container before continuing with games that put in place the symbolic value of the alternation between presence and absence (27).

Paul's compulsion to pull out his hair and to feel pain subsided and eventually disappeared when his parents started touching their child.

In denial of depressive effects, triggering the group's excitement at the nursery allowed aggressive desires to manifest. It was a noisy laugh without humor, sometimes even without comedy. The elaboration of the separation from the mother remained limited, the maternal object being little represented as a source of satisfaction.

Despite hypomanic attempts at collective laughter and the lack of resources in this small boy's symbolic activities, the transformation task of negative affect, rage, despair, and anger was not completed. Let's think of a neurosis of character in the making.

We finally quit the purely economic register of discharge to advance toward representations and a sense of humor when common mother-son laughter became possible. From motor pleasure to emotional pleasure, Paul's laughter finally led to that of his sad mother. Paul never became a clown.

I still think that even if his development went back to a more regular line. He was able to develop good language and cognitive abilities. Paul was at risk of staying in a state of narcissistic vulnerability and a tendency to use compulsive actions to manage his anxieties, as we observe in adults whose infantile trauma leaves an imprint on their capacities (28).

When he was four and was telling me a story about an abandoned child left alone in a dark forest, Paul said, “Do you remember? When I was a baby, I felt like a robot. I was like a mechanical puppet in a state of terror.”

## Conclusion

The essential contribution of the depression, of the loss of the object of satisfaction, to the construction of the psychic apparatus, is the birth of the capacity for representation, language, and symbolization.

The development of the internalized objects is precocious, and the quantity of investment attached to it depends on the pleasure of functioning in the Ego of the new activities offered by the environment, which plays a fundamental role. A child's joy in taking action and the amazement of their parents are both excellent indicators of progress after a period of initial collapse. What Geneviève Haag (29) described under the term “intracorporeal identification” as an essential sign to identify the positive evolution of a baby: when the child re-enacts with his body interactions with the mother, between integration, autoeroticism, and introjection. The adult must then interpret these experiences anchored in the body.

It seems to me that early joint therapies make it possible to reduce the defensive cleavage in the infant and to give back place and energy to the processes of identification by offering possibilities to act in the world and to experience the body in action. It is as much work with the parents to help them be more empathetic and less projective while tolerating what Winnicott calls the “ruthless cruelty” of their child as it is direct work with the baby through the corporal and interactive games.

Parent-baby psychotherapy means giving libidinal plasticity a chance.

The primary depressive collapse that I am talking about here leaves traces in the body, which keeps the trace of primary disorganizations, and in the psyche by modifying the possibilities of identification. I think that we can see here a determining influence on sensory integration disorders, now diagnosed as a nosography entity, cause serious developmental disorders.

A toddler's ability to laugh out loud or finally crack a smile is always a sign of positive development. Enthusiasm and the capacity for wonder, which are easily shared with the therapist, give the impression that treatment is going well. Serious or frightened infants and young children who are constantly worried or sad, when they cheer up, give the impression that the libidinal quality of existence has returned.

An indicator of development in toddlers is the ability to experience grief over loss, guilt for acting on aggressive impulses, and the ability to regulate these emotions. It is always important to observe the quality of the gaze, the harmony of the communication, the solidity of the motor tone, and the curiosity of a baby for the outside world. It is also necessary to evaluate the evolutionary possibilities of the family environment: can the parents have access to empathy, or do they remain in denial?

Recovery of libido begins with a renewed appreciation for life, but we must also lay the groundwork for the possibility of symbolizations.

It is time to develop research protocols highlighting the therapeutic impact of psychoanalysts' interventions, which helps us know the specific means of action for early disorders.

The contemporary world demands that analytical intervention quality be recognized by offering demonstrations that respect the scientific tradition.

Early therapies with therapeutic value and preventative potential for future narcissistic unpleasant difficulties can be provided to new parents and suffering newborns through the use of psychoanalytic thinking and an understanding of early relationship disorders.

## Data availability statement

The raw data supporting the conclusions of this article will be made available by the authors, without undue reservation.

## Ethics statement

Ethical review and approval was not required for the study on human participants in accordance with the local legislation and institutional requirements. Written informed consent to participate in this study was provided by the participants' legal guardian/next of kin.

## Author contributions

The author confirms being the sole contributor of this work and has approved it for publication.

## Conflict of interest

The author declares that the research was conducted in the absence of any commercial or financial relationships that could be construed as a potential conflict of interest.

## Publisher's note

All claims expressed in this article are solely those of the authors and do not necessarily represent those of their affiliated organizations, or those of the publisher, the editors and the reviewers. Any product that may be evaluated in this article, or claim that may be made by its manufacturer, is not guaranteed or endorsed by the publisher.
